# Timing of HIV diagnosis relative to pregnancy and postpartum HIV care continuum outcomes among Latin American women, 2000 to 2017

**DOI:** 10.1002/jia2.25740

**Published:** 2021-05-21

**Authors:** Nathaniel T Yohannes, Cathy A Jenkins, Kate Clouse, Claudia P Cortés, Fernando Mejía Cordero, Denis Padgett, Vanessa Rouzier, Ruth K Friedman, Catherine C McGowan, Bryan E Shepherd, Peter F Rebeiro

**Affiliations:** ^1^ Vanderbilt University School of Medicine Nashville TN USA; ^2^ Department of Biostatistics Vanderbilt University Medical Center Nashville TN USA; ^3^ Division of Infectious Diseases Department of Medicine Vanderbilt University Medical Center Nashville TN USA; ^4^ Vanderbilt University School of Nursing Nashville TN USA; ^5^ Fundación Arriaran y Clínica Santa María Santiago Chile; ^6^ Instituto de Medicina Tropical Alexander von Humboldt Universidad Peruana Cayetano Heredia Lima Peru; ^7^ Instituto Hondureño de Seguridad Social & Hospital Escuela Universitario Tegucigalpa Honduras; ^8^ Groupe Haitien d'Etudes du Sarcome de Kaposi et des Infections Opportunistes Port‐au‐Prince Haiti; ^9^ Instituto Nacional de Infectologia Evandro Chagas (INI) Fundação Oswaldo Cruz Rio de Janeiro Brazil

**Keywords:** HIV care continuum, HIV epidemiology, retention, vertical transmission, women, Latin America and the Caribbean (Region)

## Abstract

**Background:**

HIV incidence among women of reproductive age and vertical HIV transmission rates remain high in Latin America. We, therefore, quantified HIV care continuum barriers and outcomes among pregnant women living with HIV (WLWH) in Latin America.

**Methods:**

WLWH (aged ≥16 years) enrolling at Caribbean, Central and South America network for HIV epidemiology (CCASAnet) sites from 2000 to 2017 who had HIV diagnosis, pregnancy and delivery dates contributed. Logistic regression produced adjusted odds ratios (aOR) and 95% confidence intervals (CI) for retention in care (≥2 visits ≥3 months apart) and virological suppression (viral load <200 copies/mL) 12 months after pregnancy outcome. Cumulative incidences of loss to follow‐up (LTFU) postpartum were estimated using Cox regression. Evidence of HIV status at pregnancy confirmation was the exposure. Covariates included pregnancy outcome (born alive vs. others); AIDS diagnosis prior to delivery; CD4, age, HIV‐1 RNA and cART regimen at first delivery and CCASAnet country.

**Results:**

Among 579 WLWH, median postpartum follow‐up was 4.34 years (IQR 1.91, 7.35); 459 (79%) were HIV‐diagnosed before pregnancy confirmation, 445 (77%) retained in care and 259 (45%) virologically suppressed at 12 months of postpartum. Cumulative incidence of LTFU was 21% by 12 months and 40% by five years postpartum. Those HIV‐diagnosed during pregnancy had lower odds of retention (aOR = 0.58, 95% CI: 0.35 to 0.97) and virological suppression (aOR = 0.50, 95% CI: 0.31 to 0.82) versus those HIV‐diagnosed before.

**Conclusion:**

HIV diagnosis during pregnancy was associated with poorer 12‐month retention and virological suppression. Young women should be tested and linked to HIV care earlier to narrow these disparities.

## 1 INTRODUCTION

Over 2 million people are currently living with HIV in Latin America and the Caribbean [1]. Rates of new infections are especially high among women of reproductive age, who make up roughly half of the world’s population living with HIV [1]. It is estimated that worldwide nearly 1.5 million women living with HIV give birth each year [2], and in Latin America, between 23,000 and 37,000 pregnant women were estimated to be living with HIV as recently as 2017. [3] Fortunately, HIV screening during pregnancy is routine and treatment is frequently initiated following diagnosis.

Tremendous advancements have been made in addressing the global HIV pandemic in recent years, particularly among pregnant women. Prior to World Health Organization (WHO) “treat all” guidelines in 2015, Option B+ among pregnant and postpartum women living with HIV (WLWH) was implemented to great effect, initially in sub‐Saharan Africa and later in Latin America and the Caribbean [4, 5, 6].

Although Option B+ and subsequently “treat all” provided great promise, there are still questions regarding implementation in the postpartum period when many women exit from the HIV care cascade. Retention in care is particularly important in pregnant and lactating women because of risks associated with maternal HIV disease progression, vertical HIV transmission, paediatric HIV mortality and drug resistance. Low rates of retention, especially prevalent in resource‐limited settings, have contributed to vertical transmission rates as high as 11.4% and 13.3% in Latin America and the Caribbean, respectively, compared to 1% to 2% in high‐income countries [7, 8, 9].

We, therefore, conducted an observational study to quantify postpartum retention and virological suppression and understand barriers to engagement in care in the postpartum period within the Caribbean, Central and South America network for HIV epidemiology (CCASAnet), an HIV research network focused on better characterizing the HIV epidemic in Latin America.

### 1.1 Methods

### 1.2 Study population

CCASAnet, the Latin American region of the International epidemiology Databases to Evaluate AIDS (IeDEA), includes HIV clinic sites from seven countries (Argentina, Brazil, Chile, Haiti, Honduras, Mexico and Peru). Sites included here are in Brazil (Universidade Federal de São Paulo and Instituto Nacional de Infectologia Evandro Chagas), Chile (Fundación Arriarán), Honduras (Instituto Hondureño de Seguridad Social and Hospital Escuela Universitario) and Peru (Instituto de Medicina Tropical Alexander von Humboldt) [10].

WLWH aged ≥16 years who enrolled from 2000 to 2017, regardless of cART experience, and who had dates of HIV diagnosis, pregnancy confirmation (determined by last menstrual period, positive ultrasound, positive pregnancy test, or first presentation for prenatal care dates) and pregnancy outcomes were included. Pregnancy outcomes prior to cohort entry were not included. The study population represented between 1% and 3% of all WLWH in Latin America as of 2017 [11].

### 1.3 Outcomes and follow‐up

Retention in care, defined as ≥2 clinical visits ≥3 months apart during the first 12 months postpartum, was the primary outcome [12, 13]. For WLWH with multiple pregnancies, only the first pregnancy and postpartum period during the study period were considered. Study closure was the 95th percentile of the most recent clinic visit dates at each respective site. Women were administratively censored at the first of death, last clinic visit/CD4 count (CD4)/HIV‐1 viral load (VL) date, or study closure. Loss to follow‐up (LTFU) was defined retrospectively as having no clinical visit/CD4/VL in the 12 months preceding the study closure date.

Additional outcomes of interest included cumulative incidence of LTFU at 12 months and 5 years of postpartum [14], time to LTFU and virological suppression at 12 months postpartum. Virological suppression was determined by whether the closest VL to 12 months postpartum (±6 months) was <200 copies/mL. Primary analyses for this outcome treated those with a missing 12‐month VL as unsuppressed. Sensitivity analyses were conducted in which only those with a 12‐month VL were included.

### 1.4 Statistical analysis

Descriptives are presented as medians (interquartile range [IQR]) or percentages (frequency) for continuous and categorical variables respectively. Logistic regression models were used to quantify unadjusted and adjusted odds ratios (aOR) of postpartum retention in care and virological suppression at 12 months by *a priori* determined covariates. The exposure of interest was knowledge of HIV status at the date of pregnancy confirmation (yes vs. no). The *a priori* determined covariates included pregnancy outcome (born alive vs. all other outcomes, including miscarriage, induced abortion, stillbirth and unknown), whether AIDS was diagnosed prior to pregnancy outcome, CD4 (square‐root transformed), age, VL (log_10_‐transformed) and ART regimen at first pregnancy outcome (non‐nucleoside reverse transcriptase inhibitor [NNRTI]‐based, protease inhibitor [PI]‐based, integrase strand transfer inhibitor [INSTI]‐based/other) and CCASAnet country. AIDS prior to pregnancy outcome was defined as the earliest indication of an AIDS‐defining event or diagnostic staging of AIDS compared to the pregnancy outcome date. Both univariate and multivariable models (complete‐case and multiply‐imputed with 10 imputation replications to account for missing data) were fit, and univariate models were fit on all covariates. Multivariable models included only *a priori* identified covariates, including likely confounders, and CCASAnet country due to limited degrees of freedom.

Unadjusted and adjusted relative hazards (aHR) of LTFU by the exposure of interest were assessed using Cox regression, with the multivariable model stratified by site (allowing separate inter‐site baseline hazards). Covariates included in Cox models were the same as those in logistic regression models.

Ethical clearance for the study was obtained by local institutional review boards (IRBs) as well as the Vanderbilt University School of Medicine IRB; a waiver of consent was obtained for participation in this analysis. All study activities complied with the Declaration of Helsinki.

## 2 RESULTS

A total of 579 WLWH who were pregnant while receiving HIV care from 2000 to 2017 were included: 166 from Brazil, 93 from Chile, 85 from Honduras and 235 from Peru. Median age at delivery was 29 (IQR 24, 34) years, median CD4 at delivery was 396 (IQR 250, 581) cells/µL and 107 (18%) women had an AIDS‐defining event prior to their first pregnancy outcome. The median follow‐up after the first pregnancy outcome was 4.34 (IQR 1.91, 7.35) years.

Among participants, 459 (79%) were HIV‐diagnosed before the date of their pregnancy confirmation. Women in Brazil had the highest percentage of HIV diagnoses prior to their first pregnancy (93%); women in Peru had the lowest (63%). At the time of pregnancy confirmation, 280 (48%) had already started cART. At the time of delivery, 447 (77%) of women were taking cART: 128 (22%) on NNRTI‐based, 278 (48%) on PI‐based, 15 (3%) on INSTI‐based and 26 (4%) on other regimens; 132 (23%) were not on cART. A total of 509 (88%) of the pregnancies resulted in live births (Table 1).

**Table 1:** Descriptive statistics by the timing of HIV diagnosis relative to the first pregnancy in the cohort

	HIV diagnosed after pregnancy confirmation	HIV diagnosed prior to pregnancy confirmation	Combined
	N = 120	N = 459	N = 579
Age (years)	26 (22, 30)	30 (25, 35)	29 (24, 34)
Calendar year	2013 (2010, 2016)	2013 (2009, 2015)	2013 (2009, 2015)
Pregnancy outcome			
Born alive	118 (98)	391 (85)	509 (88)
Induced abortion	1 (1)	17 (4)	18 (3)
Spontaneous abortion	1 (1)	41 (9)	42 (7)
Stillbirth	0 (0)	7 (2)	7 (1)
Unknown	0 (0)	3 (0)	3 (1)
Days from pregnancy confirmation to pregnancy outcome	171 (109, 214)	176 (102, 223)	175 (103, 221)
Index pregnancy occurring prior to cART initiation	98 (82)	182 (40)	280 (48)
On cART at pregnancy outcome	74 (62)	373 (81)	447 (77)
cART regimen at pregnancy outcome			
NNRTI‐based regimen	24 (32)	104 (28)	128 (29)
PI‐based regimen	36 (49)	242 (65)	278 (62)
INSTI‐based regimen	9 (12)	6 (2)	15 (3)
Other cART regimen	5 (7)	21 (6)	26 (6)
Year of HIV diagnosis	2013 (2010, 2015)	2008 (2005, 2012)	2009 (2005, 2013)
Number of previous pregnancies			
0	119 (99)	413 (90)	532 (92)
1	1 (1)	41 (9)	42 (7)
≥2	0 (0)	5 (1)	5 (1)
Probable route of infection			
Heterosexual contact	119 (99)	420 (92)	539 (93)
Other/unknown	1 (1)	39 (8)	40 (7)
CD4 at pregnancy outcome (cells/µL)	347 (190, 523)	417 (256, 598)	396 (250, 581)
Missing	11 (9)	24 (5)	35 (6)
HIV‐1 RNA at pregnancy outcome (log_10_ copies/mL)	2.3 (2.3, 3.2)	2.3 (2.3, 2.3)	2.3 (2.3, 2.3)
Undetectable (<200 copies/mL)	53 (57)	260 (80)	313 (75)
Missing	27 (23)	133 (29)	160 (28)
Prior AIDS	8 (7)	99 (22)	107 (18)
Country of clinical care			
Brazil	11 (9)	155 (34)	166 (29)
Chile	13 (11)	80 (17)	93 (16)
Honduras	8 (7)	77 (17)	85 (15)
Peru	88 (73)	147 (32)	235 (41)

Numbers presented as count (%) or else as median (interquartile range) for all continuous covariates to avoid influence in measures of central tendency due to normality violations or skew. cART, combination antiretroviral therapy; NNRTI, non‐nucleoside reverse transcriptase inhibitor; PI, protease inhibitor; INSTI, integrase strand transfer inhibitor.

Within our study population, 445 (77%) women were retained in care in the first year postpartum. Women in Brazil had the highest level of retention (90%) and Chile the lowest (56%). There was evidence of an association between the timing of HIV diagnosis relative to pregnancy and 12‐month retention outcomes in this cohort. In univariate analysis, those diagnosed with HIV after pregnancy confirmation had 48% lower odds of being retained (*p* = 0.003; Table 2) versus those diagnosed before. After adjusting for pregnancy outcome, AIDS prior to delivery, CD4 and age at delivery and study site, this association was slightly attenuated (aOR = 0.58, 95% confidence interval [CI]: 0.35 to 0.97, *p* = 0.04). Pregnancy outcomes other than live birth and AIDS diagnosis prior to birth were associated with increased odds of being retained 12 months after pregnancy outcome (Table 2).

**Table 2:** Odds ratios (and 95% confidence intervals) for retention in care during the first year after the end of the pregnancy

	Univariate			Imputed Multivariable		
	OR	95% CI	*p*	OR	95% CI	*p*
HIV diagnosis after pregnancy confirmation	0.52	(0.33, 0.80)	0.003	0.58	(0.35, 0.97)	0.04
Not a live birth	5.40	(1.93, 15.13)	0.001	2.72	(0.91, 8.13)	0.07
AIDS prior to pregnancy outcome	4.51	(2.13, 9.53)	<0.001	3.77	(1.71, 8.31)	<0.001
CD4 at pregnancy outcome (per 5 square‐root cells/µL)	1.00	(0.84, 1.18)	0.96	0.84	(0.69, 1.03)	0.09
Age at delivery (per 5 years)	1.22	(1.04, 1.42)	0.02	1.12	(0.93, 1.33)	0.22
Site of care0.010						
Brazil (reference)	1.00			1.00		<0.001
Chile	0.14	(0.08, 0.28)		0.18	(0.09, 0.36)	
Honduras	0.29	(0.15, 0.58)		0.24	(0.11, 0.52)	
Peru	0.40	(0.22, 0.72)		0.55	(0.28, 1.05)	
HIV‐1 RNA at baseline (per log_10_ copies/mL)	0.68	(0.51, 0.91)	< 0.001			
cART regimen at pregnancy outcome			<0.001			
NNRTI‐based (reference)	1.00					
PI‐based	1.46	(0.83, 2.55)				
INSTI‐based/Other	0.50	(0.22, 1.10)				
Not on cART	0.29	(0.16, 0.50)				

Baseline was the date of pregnancy outcome. cART, combination antiretroviral therapy; CI, confidence interval; INSTI, integrase strand transfer inhibitor; NNRTI, non‐nucleoside reverse transcriptase inhibitor; OR, Odds ratio; PI, protease inhibitor.

**Figure 1:**
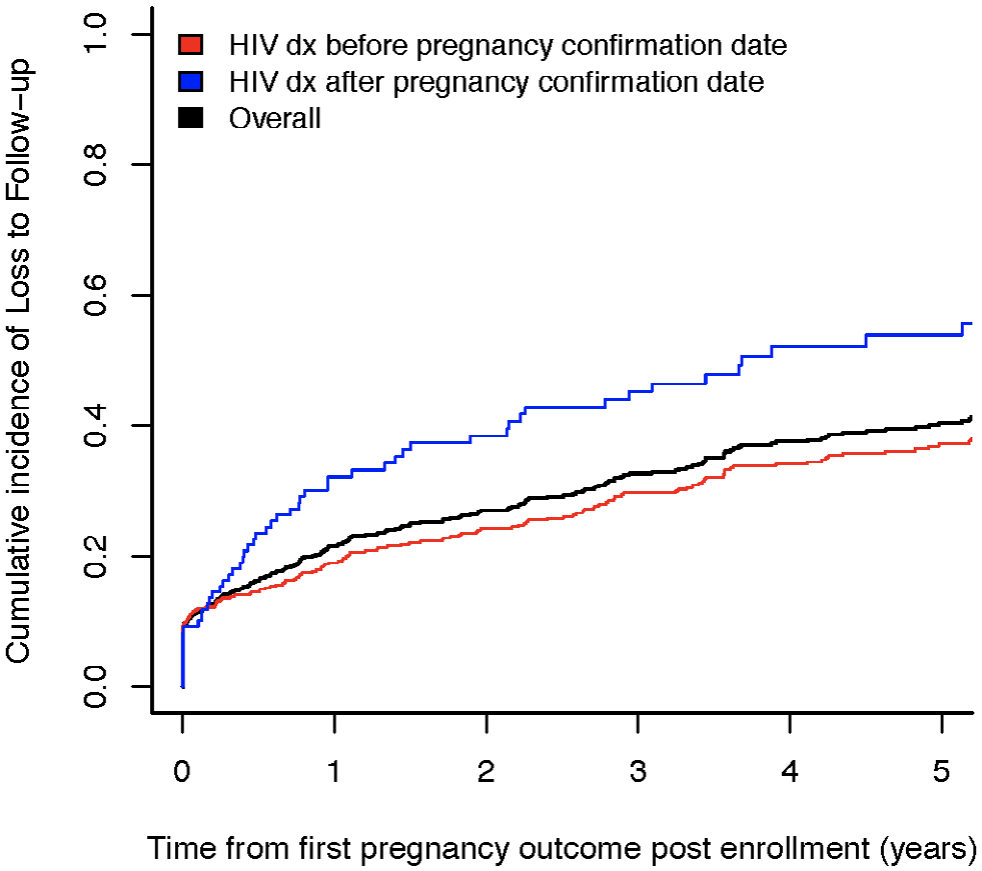
Cumulative incidence of loss to follow‐up (LTFU) among women enrolled at CCASAnet sites, stratified by the relative timing of HIV diagnosis date with respect to pregnancy confirmation date.

The cumulative incidence of LTFU among women after pregnancy was 21% by 12 months and 40% by 5 years; 81 participants had more than one pregnancy outcome after cohort entry, 20 women died in the post‐enrolment period and 64 women were LTFU at the end of pregnancy itself (Figure 1).

The hazard of LTFU was 69% higher for women diagnosed with HIV during pregnancy (*p* < 0.001). The increased hazard of LTFU among those diagnosed with HIV during pregnancy versus those diagnosed before remained after adjusting for covariates (aHR = 1.50, 95% CI: 1.08 to 2.09, *p* = 0.02). Similar to the retention analysis, pregnancy outcomes other than live birth and AIDS diagnosis prior to pregnancy outcome were associated with decreased hazards of being LTFU. Older age was also associated with a decreased risk of LTFU (Table 3).

**Table 3:** Hazard ratios (and 95% confidence intervals) for loss to follow‐up within first year after delivery. Analyses are stratified by study site

	Univariate			Imputed Multivariable		
	HR	95% CI	*p*	HR	95% CI	*p*
HIV diagnosis after pregnancy confirmation	1.69	(1.27, 2.25)	<0.001	1.50	(1.08, 2.09)	0.02
Not a live birth	0.33	(0.19, 0.55)	<0.001	0.66	(0.37, 1.17)	0.16
AIDS prior to pregnancy outcome	0.55	(0.39, 0.78)	<0.001	0.68	(0.46, 0.99)	0.04
CD4 at pregnancy outcome (per square‐root cells/µL)	0.95	(0.85, 1.05)	0.30	1.03	(0.93, 1.14)	0.56
Age at pregnancy outcome (per 5 years)	0.84	(0.76, 0.93)	<0.001	0.89	(0.80, 1.00)	0.04
HIV1‐RNA at baseline (per log_10_ copies/mL)	1.16	(0.98, 1.37)	0.09			
cART regimen at pregnancy outcome			<0.001			
NNRTI‐based	1					
PI‐based	0.89	(0.63, 1.25)				
INSTI‐based/Other	1.75	(1.03, 2.99)				
Not on cART	1.66	(1.16, 2.40)				

Cox models stratified by site of care. cART, combination antiretroviral therapy; CI, confidence interval; HR, hazard ratio; INSTI: integrase strand transfer inhibitor; NNRTI, non‐nucleoside reverse transcriptase inhibitor; PI, protease inhibitor.

The proportion of women with confirmed virological suppression closest to 12 months postpartum was 45%. Those diagnosed with HIV during pregnancy (aOR = 0.50, 95% CI: 0.31 to 0.82, *p* = 0.006) and those with higher baseline VL had lower odds of being virologically suppressed 12 months after pregnancy outcome compared with those diagnosed before pregnancy (Table 4). In contrast, those whose pregnancy outcome was stillbirth, induced abortion or miscarriage and older women, had higher odds of virological suppression (Table 4). These results were substantively similar when excluding women with missing VL data.

**Table 4:** Odds ratios (95% confidence intervals) for viral suppression 12 months after pregnancy outcome

	Univariate			Multivariable		
	OR	95% CI	*p*	OR	95% CI	*p*
HIV diagnosis after pregnancy confirmation	0.45	(0.29, 0.70)	<0.001	0.50	(0.31, 0.82)	0.006
Not a live birth	2.62	(1.53, 4.46)	<0.001	1.62	(0.90, 2.90)	0.11
AIDS prior to first pregnancy outcome	1.84	(1.20, 2.80)	0.005	1.43	(0.90, 2.27)	0.13
CD4 at pregnancy outcome (square root transformed, 200‐unit increase)	1.12	(0.97, 1.28)	0.12	1.02	(0.87, 1.19)	0.84
Age at pregnancy outcome (per 5 years)	1.43	(1.25, 1.64)	<0.001	1.39	(1.19, 1.61)	<0.001
Site of care			<0.001			<0.001
Brazil (reference)	1.00			1.00		
Chile	0.34	(0.20, 0.58)		0.43	(0.24, 0.78)	
Honduras	0.50	(0.29, 0.85)		0.53	(0.29, 0.96)	
Peru	0.71	(0.48, 1.06)		1.18	(0.74, 1.87)	
HIV‐1 RNA at baseline (per log_10_ copies/mL)	0.54	(0.40, 0.73)	<0.001			
cART regimen at pregnancy outcome			0.10			
NNRTI‐based	1.00					
PI‐based	1.12	(0.74, 1.71)				
INSTI‐based/Other	0.53	(0.26, 1.11)				
Not on cART	0.40	(0.24, 0.67)				

cART, combination antiretroviral therapy; CI, confidence interval; INSTI, integrase strand transfer inhibitor; NNRTI, non‐nucleoside reverse transcriptase inhibitor; OR, odds ratio; PI, protease inhibitor.

## 3 DISCUSSION

Retention in HIV care during the first year postpartum in this Latin American group of WLWH was relatively high compared to similar populations in high‐income settings. Furthermore, the timing of an HIV diagnosis prior to pregnancy was strongly associated with 12‐month retention outcomes postnatally in this cohort, whereas those who were HIV‐diagnosed during pregnancy had lower odds of virological suppression 12 months after the pregnancy outcome. Poorer clinical conditions such as AIDS diagnosis prior to pregnancy outcome and pregnancy outcomes aside from live birth were associated with improved retention in care and reduced likelihood of being LTFU. In addition, pregnancy outcomes other than live birth were associated with improved virological suppression at 12 months postpartum.

We found retention in care during the first year after pregnancy outcome in our Latin American HIV cohort (77%) to be similar or higher compared to several sub‐Saharan African (71% in South Africa, 77% in Malawi, 42% in Mozambique) and United States (37% in Jackson, Mississippi, 39% in Philadelphia, 24% in New York) cohorts that have been well described [15, 16]. In these studies, the vast majority of women either silently transferred care to other clinics or, particularly in the United States, dropped out of clinical care entirely for periods longer than one year. It is possible that these same mobility, migration and transfer‐of‐care patterns undergird observations of retention and LTFU in our own study population, though we are not currently able to quantify them. Among the six sites located in four countries that we investigated, those in Brazil had the highest proportion retained (90%). Τhis may be related to the high percentage of HIV diagnoses prior to first pregnancy after enrolment, which implies that linkage‐to‐care was successful prior to pregnancy. It may also be due in part to Brazil’s national efforts in the last two decades to implement health policies that guarantee free access to HIV testing, cART and coverage of prenatal and birth care resources. In fact, it is estimated that, in Brazil, 98.7% of pregnant women receive prenatal care [17]. That said, country‐level estimates for UNAIDS indicators such as cART coverage among women ≥15 years old in the very same countries represented in our study range quite widely from 32% in Chile, to approximately 65% in Brazil, Honduras and Peru, meaning the programmatic context of our study population may also have varied from one site to the next. [11]

The presence of numerous individual‐level factors has likewise been noted to influence significant disparities in HIV care outcomes of young postpartum WLWH. Several studies conducted in sub‐Saharan Africa demonstrated that this group was much more likely to be LTFU compared to other women [18]. One of the strongest predictors for LTFU in the postpartum period is lack of retention in the preconception period, which has been shown in other settings to increase the likelihood of being LTFU postpartum threefold [16]. Additionally, lower odds of retention in care postnatally, alongside poor cART adherence and lower rates of viral suppression at delivery, are associated with HIV diagnosis during pregnancy [19]. This highlights the importance of diagnosing and engaging young WLWH in HIV care with access to cART early.

Other factors found to be associated with a higher risk of LTFU in prior work are unemployment, lack of basic needs such as electricity in the home [18], substance use, lower educational attainment [20] and social factors that include fear of disclosure, lack of social support and negative experiences with providers [21]. It has also been shown that this population remains vulnerable to suboptimal health outcomes and lack of clinical care due to increased responsibilities, emotional stress and stigma related to HIV disclosure during pregnancy [21]. It remains to be investigated whether infant care is a specific barrier to engagement in HIV care, but, women who do not disclose their HIV status to their partner have been shown to be more likely to delay HIV care and less likely to be virologically suppressed at delivery and retained in the postpartum period [16]. In our own study, we found large differences in clinical retention in regard to the relative timing of HIV diagnosis. Viral suppression also appeared to be influenced by whether HIV was diagnosed during pregnancy or not. One possible explanation is that earlier knowledge of HIV diagnosis allows patients and providers to build rapport and navigate treatment decisions together. Nevertheless, more qualitative and quantitative work should be done to investigate barriers to retention and viral suppression for those diagnosed with HIV during pregnancy in order to prevent vertical HIV transmission.

Evaluations of retention in care after HIV diagnosis in low‐ and lower‐middle‐income settings also currently suggest that youth and general good health, characteristics of most women of reproductive age, are associated with worse care continuum outcomes [22, 23, 24, 25]. Studies from several sub‐Saharan African countries found higher risks of LTFU among women who were younger, had a higher CD4, and were pregnant [23, 26, 27]. Similarly, women who initiated cART at CD4 counts ≥300 cells/μL have been found to be at an increased risk of LTFU in the first 24 months [28]. Lower retention in this population, and contrariwise, higher retention among women with poor health or adverse pregnancy outcomes, may be due to diminished care‐seeking behaviour when individuals feel relatively healthy or do not immediately feel physical benefits from cART. These individual factors that characterize young and otherwise healthy WLWH at risk of LTFU may also synergize with structural factors, in that resource‐limited clinics may be more likely to focus on treating acutely ill HIV patients and those with advanced immunosuppression; they may lack funding to support programmes targeted towards retaining healthier patients, putting those individuals at an elevated risk of disengagement from care. Furthermore, there is evidence that psychosocial challenges such as social isolation and stigma, as well as lack of peer engagement, may diminish care engagement among young postpartum women. [21, 29]

The totality of the evidence, then, suggests that early engagement in HIV care may be an important protective practice in preventing postpartum losses from HIV care. Coverage of HIV testing in women before the first pregnancy should be improved and be monitored. A study conducted in the United States identified predictive factors for long‐term retention (defined as retention ≥12 months postpartum) and found that women who engaged in care early (HIV care within 90 days postpartum) were 11 times more likely to be retained long term [16]. One way to ensure early engagement is integrating HIV care for the mother with paediatric care for the child. This can occur by delivering care at the same site or even within the same visit and has been shown to be effective in increasing retention and viral suppression in countries like South Africa [30]. Another strategy shown to be effective for increasing patient engagement is alleviating financial burdens by providing free access to cART [31] and transportation subsidies [21]. Recent trials in Nigeria, Zimbabwe and Malawi demonstrated that peer support interventions are efficacious at improving retention in young mothers living with HIV [21]. Another key component in improving retention rates that has been described in the United States is the utilization of case management, which can help mitigate barriers to healthcare such as lack of transportation, housing and food insecurities [16]. Counselling and education are very important as well and should be individualized especially when caring for patients with higher CD4 that are at an elevated risk of leaving care. Moreover, finding creative methods of care delivery and HIV screening in the community as opposed to traditional medical facilities may help improve virological outcomes. [32, 33, 34] It is therefore imperative that we rethink resource allocation when considering young WLWH in order to improve morbidity and mortality among women and paediatric HIV‐exposed‐uninfected and HIV‐positive populations.

Our study did have important limitations. First, we may have measurement errors in covariates due to harmonizing clinical data collected differently at multiple sites. However, it is also an important strength of this study that pooling clinical cohorts across multiple sites in our region resulted in a large study sample and a diversity of clinical and patient characteristics. We also realize that WLWH in these clinical cohorts may not represent all WLWH in settings with similar sociodemographic profiles, so our results may have limited generalizability. Moreover, it may be challenging to compare outcomes directly with prior studies because of subtle differences in definitions of key variables (e.g. LTFU, which is defined here as 12 months without care before study closure, but elsewhere, has been classified as the first 3‐, 6‐ or 12‐month gap in care). [20, 35] Other potential barriers to inference include unmeasured confounding, potential selection biases (e.g. if HIV testing and care referral practices differed between women diagnosed during and before pregnancy, or the late presentation and HIV diagnosis of some women delayed ART initiation), and changes in clinical and programme management, such as guidelines for cART use during pregnancy and universal treatment (under WHO guidance from 2015), over an admittedly long study period spanning nearly 20 years. [4, 6] Despite these potential shortcomings, we believe we have used a rich source of relevant data and analysed it using robust methods, including adjustment for important confounders, to arrive at inferences that may be of great interest to non‐governmental organizations, policy makers, public health practitioners and funding agencies in light of the rise in women’s health awareness and lack of data regarding pregnancy and HIV in Latin America. Furthermore, this work may support and inform future study designs that would support a causal relationship between similarly identified factors and retention and virological outcomes.

## 4 CONCLUSION

Our findings highlight the importance of revisiting treatment and care guidelines to improve the quality of HIV care delivered to pregnant women and women of reproductive age. The complex nature of numerous obstacles women must overcome to receive stable HIV care reflects the public health challenges we will need to address on a clinical, individual and social level. We must also continue to collect data that will inform the development of innovative models and effective interventions for improving ART programmes around the world. If our goal is to reduce HIV morbidity and mortality and mitigate HIV transmission, then we must learn to engage women of child‐bearing age and postpartum women in a way that is tailored to their needs and the needs of their families.

## COMPETING INTEREST

All authors declare no conflicts beyond the funding listed above (money paid to their respective institutions).

## AUTHORS’ CONTRIBUTIONS

CAJ: curated/managed the data, conducted statistical analyses, helped interpret the findings and critically edited the final manuscript. CPC, FMC, DP, VR and RKF: helped design the study, contributed data and critically edited the final manuscript. NTY, KC, CCM, BES and PFR: helped design the study, helped draft the manuscript, helped interpret the findings and critically edited the final manuscript. All authors read and approved the final manuscript.
